# Impact of HIV-1 capsid polymorphisms on viral infectivity and susceptibility to lenacapavir

**DOI:** 10.1128/mbio.00187-25

**Published:** 2025-04-17

**Authors:** Derek Hansen, Matthew R. Hendricks, Silvia Chang, Arthur Cai, Jason K. Perry, Thomas Aeschbacher, Ross Martin, Tomas Cihlar, Stephen R. Yant

**Affiliations:** 1 Department of Research Discovery Virology, Gilead Sciences, Inc., Foster City, California, USA; 2 Department of Research Clinical Virology, Gilead Sciences, Inc., Foster City, California, USA; 3 Department of Research Structural Biology and Chemistry, Gilead Sciences, Inc., Foster City, California, USA; University of Pittsburgh School of Medicine, Pittsburgh, Pennsylvania, USA; University of Pittsburgh School of Medicine, Pittsburgh, Pennsylvania, USA

**Keywords:** polymorphism, antiretroviral resistance, lenacapavir, HIV, viral fitness

## Abstract

Lenacapavir (LEN) is a first-in-class capsid (CA) inhibitor for the treatment and prevention of HIV-1 infection. While LEN has shown potent antiviral activity across all major HIV-1 subtypes, the impact of existing HIV-1 CA sequence diversity on the activity of LEN remains to be determined. Here, we identified natural polymorphisms within the LEN-binding site and assessed each for their impact on viral infectivity and susceptibility to LEN. Using a co-crystal structure of LEN in complex with a CA hexamer, we identified 29 binding site residues within five angstroms of LEN and analyzed each for naturally occurring polymorphisms across a multiclade collection of >10,000 unique HIV-1 *gag* sequences. Eleven of these CA residues, including five (M66, Q67, K70, N74, and A105) previously associated with LEN resistance when mutated, were invariant across these sequences. The remaining 18 residues showed one or more substitutions with a ≥0.5% prevalence for a total of 54 CA polymorphisms. When introduced as site-directed mutants (SDMs) in an NL4.3-based reporter virus and evaluated for infectivity and drug susceptibility in MT-4 cells, 74% (40/54) showed impaired infectivity (0.01%–77% of wild type), with 96% (46/48) exhibiting minimal change (less than threefold) in susceptibility to LEN. While CA substitutions L56V and N57H conferred high-level resistance to LEN (72- and 4,890-fold, respectively), both variants showed diminished replication capacity in primary T-cells relative to the wild-type virus. Collectively, these results indicate that existing CA natural HIV-1 sequence diversity within the LEN-binding site is rare and should minimally impact LEN efficacy in treatment-naïve individuals.

**IMPORTANCE:**

HIV-1 capsid protein mediates multiple essential functions throughout the viral replication cycle, making it an attractive target for therapeutic intervention. Lenacapavir (LEN), a first-in-class HIV-1 capsid inhibitor, is being evaluated as a long-acting option in multiple ongoing clinical studies for HIV treatment and prevention. Twice-yearly lenacapavir is approved in multiple countries for the treatment of adults with multi-drug-resistant HIV-1 in combination with other antiretrovirals, and its investigational use for pre-exposure prophylaxis has shown 99.9%–100% efficacy in preventing HIV infection among a broad and geographically diverse range of study participants. In this report, we investigated how HIV-1 sequence diversity within the LEN binding site may impact virus replication capacity and sensitivity to LEN. Our data demonstrate high capsid sequence conservation across a large and diverse collection of HIV-1 variants, with the majority of naturally occurring capsid polymorphisms having a detrimental effect on viral infectivity and minimal impact on susceptibility to LEN.

## INTRODUCTION

The HIV type 1 (HIV-1) pandemic has spanned more than four decades and remains a major public health challenge that has claimed over 42 million lives to date, with an estimated 40 million people currently living with HIV worldwide (1). Although a diagnosed HIV-1 infection was previously associated with a high mortality rate, most people with HIV (PWH) can now reach their full life expectancy due to the development of multiple approved highly active antiretroviral combination therapies (2–4). While current standard-of-care once daily oral HIV treatment medications are both safe and highly effective, the lifelong requirement for near-perfect adherence to prevent the emergence of drug resistance in PWH remains a significant challenge. As such, there remains a high unmet medical need for the development of new antiretroviral agents suitable for less frequent dosing (i.e., long-acting) to support enhanced convenience, reduced emotional burden/stigma, and improved health outcomes via greater adherence.

The HIV-1 capsid (CA) protein is the chief structural component of the viral capsid core lattice, which is arranged as a fullerene-like cone composed of approximately 250 CA hexamers and precisely 12 CA pentamers. HIV-1 CA plays multiple roles throughout the viral replication cycle, including those essential for the proper formation, optimal stability, and intracellular trafficking of the viral core through the cytoplasm, nuclear pore complex (NPC), and nuclear compartment (5–22). Considering the many essential roles CA plays during HIV-1 replication, together with its extreme genetic fragility (5, 6, 23), the viral capsid has emerged as an attractive target for therapeutic intervention (24–28).

Lenacapavir (LEN, formerly GS-6207) is a potent, first-in-class, multistage HIV-1 CA inhibitor that binds at the interface formed between two adjacent capsid subunits within the viral capsid lattice (29, 30). LEN exhibits distinct, highly potent antiviral activities during both the early and late stages of HIV-1 replication. During the infection of target cells, LEN binds to and hyper-stabilizes mature HIV-1 capsid cores and blocks their nuclear import, thereby preventing the formation of a stable integrated DNA reservoir (29–32). During the late stages of HIV-1 replication, LEN also interferes with proper virion maturation to produce non-infectious virions with aberrant viral capsids (29, 30, 33). In addition, the LEN-binding site on the capsid partially overlaps with that of multiple phenylalanine-glycine (FG)-motif-containing host cofactors essential for HIV replication, including the cytoplasmic protein Sec24C, the NPC basket protein nucleoporin 153 (Nup153), and the nuclear trafficking protein cleavage, and polyadenylation specificity factor 6 (CPSF6) (29, 34–39). In this manner, LEN binding to the capsid also interferes with the normal binding of these host cofactors, which can impair nuclear import by Nup153 (29, 30) and diminish proper nuclear capsid core trafficking to transcriptionally active sites on host cell chromatin by CPSF6 (40, 41). LEN’s novel mode of action helps make it an effective inhibitor of both wild-type (WT) HIV-1 and variants with resistance to other classes of antiretroviral agents (29). In addition, the picomolar potency of LEN, combined with its exceptionally low *in vivo* systemic clearance and slow-release kinetics, makes it an attractive long-acting antiretroviral agent amenable to a variety of dosing frequencies, ranging from once-daily and once-weekly oral up to twice-yearly injectable (42, 43). Accordingly, LEN is now under extensive clinical development as a foundation for future investigational long-acting HIV therapies in PWH and for HIV prevention in people who would benefit from pre-exposure prophylaxis (PrEP) (44–47).

In a proof-of-concept phase 1 monotherapy study, a single subcutaneous administration of a long-acting formulation of LEN-supported therapeutic drug concentrations in the plasma of both healthy participants (GS-US-200-4070) and people with untreated HIV-1 infection (NCT03739866) for at least 6 months (29). Moreover, when tested in combination with an optimized background regimen in a phase 2/3 trial (CAPELLA, NCT04150068), twice-yearly subcutaneous LEN demonstrated a mean plasma HIV-1 RNA decline of −2.10 log_10_ copies per mL after 14 days of functional monotherapy and maintained high rates of virologic suppression (82%, HIV-1 RNA < 50 copies/mL) over 104 weeks in people with multidrug-resistant HIV-1 infection (48–50), leading to its regulatory approval as a component of a combination HIV-1 treatment regimen for use in heavily treatment-experienced PWH. In addition to its use in HIV treatment, LEN has shown tremendous promise as an investigational long-acting monotherapy agent for HIV prevention among a broad, geographically diverse range of study participants, with twice-yearly subcutaneous LEN reducing HIV infection by 100% and 96% compared with the background HIV incidence in the phase 3 PURPOSE 1 (NCT04994509) and PURPOSE 2 (NCT04925752) studies, respectively (44, 46).

In cell culture, LEN and its close analog GS-CA1 select for emergent HIV-1 variants encoding one or more CA substitutions within the LEN-binding site (L56I, N57S, M66I, Q67H, K70R/N, N74D/S, and/or T107N) that either alone or in combination confer reduced susceptibility to LEN (29, 51). In the phase 2/3 trial of twice-yearly subcutaneous LEN used in combination with an optimized oral background regimen for the treatment of multidrug-resistant HIV-1 infection, 19% (14/72) of study participants developed one or more substitutions within the LEN-binding site over 104 weeks, including M66I (detected in six participants), Q67H/K/N (in eight participants), K70H/N/R/S (in seven participants), N74D/H/K (in five participants), A105S/T (in five participants), and T107A/C/N/S (in six participants), primarily due to inadequate adherence to the optimized background regimen and/or functional LEN monotherapy (48–50). In addition, LEN given subcutaneously or orally in combination with currently approved daily antiretrovirals maintained high rates of virologic suppression (87%) in a phase 2 trial of people with untreated HIV-1 infection (CALIBRATE, NCT04143594) with 2% (3/157) of study participants developing two LEN resistance-associated mutations (RAMs; Q67H and K70R) in CA after 80 weeks of therapy (52).

Besides the RAMs observed in CA with the use of LEN both *in vitro* and in PWH, little is known regarding CA polymorphisms that might be in circulating HIV strains and how any, particularly those located within the LEN-binding pocket, might affect the HIV-1 replication capacity and susceptibility to LEN. Here, we analyzed capsid sequence diversity across HIV-1 group M subtypes from >10,000 LEN treatment-naïve individuals. Capsid polymorphisms within the LEN-binding site were then evaluated as site-directed HIV-1 mutants and individually assessed for their impact on viral infectivity and susceptibility to LEN.

## RESULTS

### Identification of HIV-1 CA protein residues near bound lenacapavir

**Fig 1:**
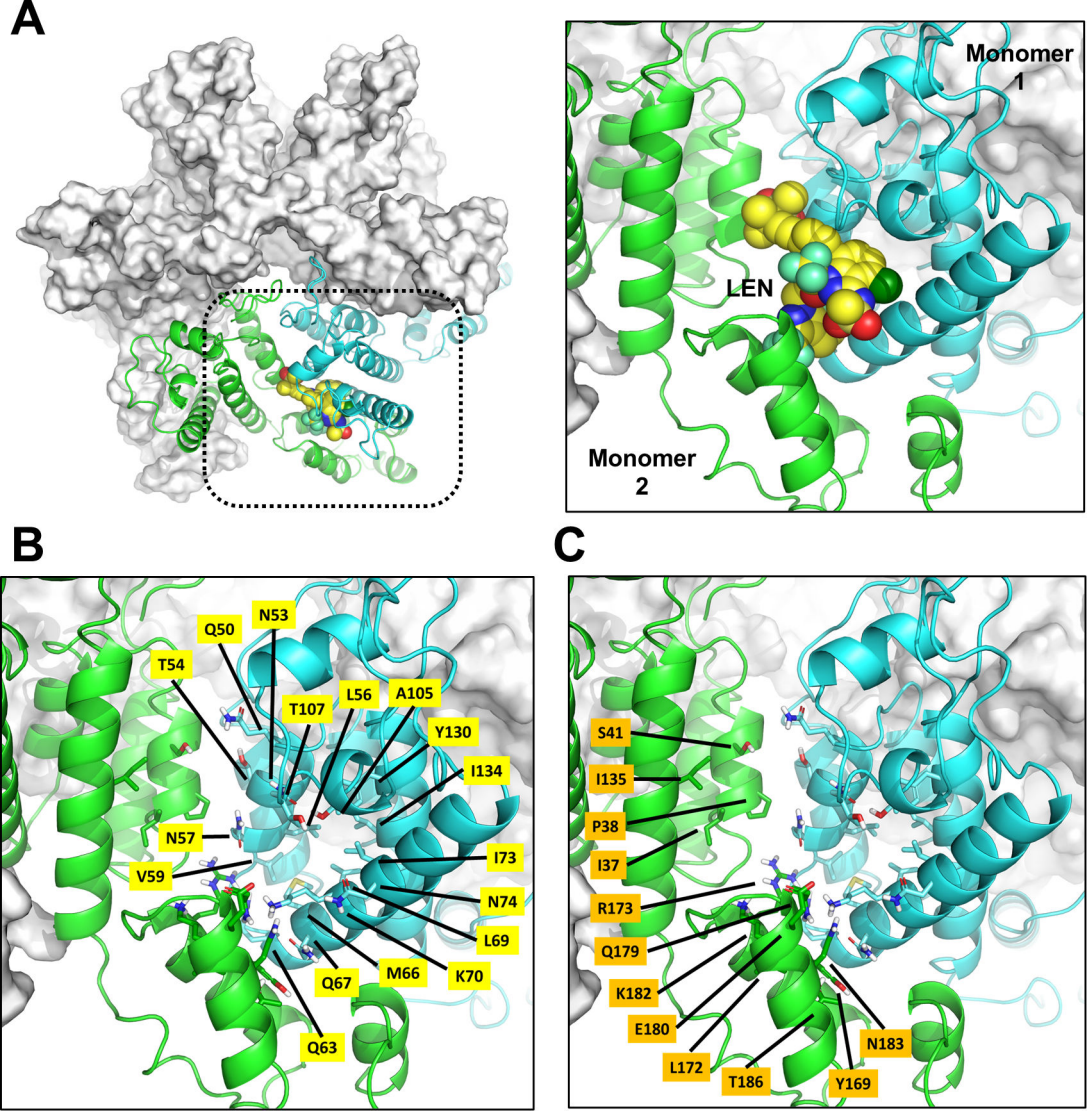
HIV-1 CA residues within five angstroms of LEN. (A) LEN bound to cross-linked CA hexamer protein. Left, top surface view of CA hexamer (gray) with LEN (yellow) bound at the interface formed between one CA monomer (cyan ribbon) and its adjacent CA monomer (green ribbon). Right, close-up view of LEN in its binding site. (B and C) Residues defining the LEN-binding site in adjacent CA monomers. CA amino acid side chains within five angstroms of LEN are highlighted in yellow and orange for CA monomer 1 and 2, respectively.

To determine which CA residues have the greatest potential to impact HIV susceptibility to LEN, a co-crystal structure of LEN bound to recombinant cross-linked capsid hexamer protein (PDB 6V2F) (29) was used to identify all amino acid residue side chains within five angstroms of the bound inhibitor. In total, 29 residues were identified within or adjacent to the site where LEN binds at the interface formed between two adjacent CA monomers (Fig. 1A). Of these, 17 amino acids (Q50, N53, T54, L56, N57, V59, Q63, M66, Q67, L69, K70, I73, N74, A105, T107, Y130, and I134) were contributed by the N-terminal domain of the CA monomer directly bound by LEN (Fig. 1B). The remaining 12 residues of interest were localized to the adjacent CA monomer, either as part of its N-terminal domain (I37, P38, S41, and I135) or C-terminal domain (Y169, L172, R173, Q179, E180, L182, N183, and T186; Fig. 1C). This set of 29 residues captures all CA amino acids in direct contact with LEN, as well as those in which a mutation could introduce a direct contact with LEN. It also captures second-shell CA amino acids with the highest probability of altering the LEN-binding site. While we cannot rule out that more distant residues could potentially influence LEN binding either directly or via long-range indirect effects, such residues were outside the scope of this study.

### HIV-1 capsid sequence characteristics

To compile a sufficiently diverse data set for assessment of HIV-1 CA sequence variation, 23,890 full-length *gag* sequences were downloaded from the Los Alamos HIV public sequence database (June 2023 build) and combined with 825 full-length unique *gag* sequences obtained from Gilead-sponsored clinical trial samples from study participants never previously exposed to LEN. The public data set was curated to remove duplicate sequences by keeping only the first sequence with each unique patient ID, resulting in a total of 9,232 individual sequences, which when combined with the internal data set brought the total number of unique *gag* sequences included in this analysis to 10,057. These combined sequences spanned six distinct subtypes (A1, B, C, D, F1, and G) and two circulating recombinant forms (CRF01_AE and CRF02_AG; Table 1). Globally, subtypes C, B, and A are responsible for 50%, 12%, and 11% of all HIV-1 infections, respectively, followed by CRF02_AG (7%) and CRF01_AE (5%) (53). Among the combined *gag* sequence data set analyzed in this study, subtype B was the most represented clade at 56.6% (5,689/10,057) of total sequences followed by clade C and subtype CRF01_AE at 15.5% and 14.2% of sequences, respectively. The five remaining subtypes comprised a total of 14.7% of unique *gag* sequences, with 4.9%, 4.3%, 3.4%, 1.3%, and 0.9% of sequences coming from subtypes A1, D, CRF02_AG, G, and F1, respectively.

**TABLE 1:** Summary of curated unique HIV-1 group M CA sequences

HIV-1 subtype	Public (*n* = 9,232)	Clinical (*n* = 825)	Total sequences (*n* = 10,057)
B	4,970	719	5,689
A1	485	4	489
C	1,543	15	1,558
D	376	54	430
F1	88	1	89
G	125	3	128
CRF01_AE	1,308	20	1,328
CRF02_AG	337	9	346

### Capsid conservation analysis

**Fig 2:**
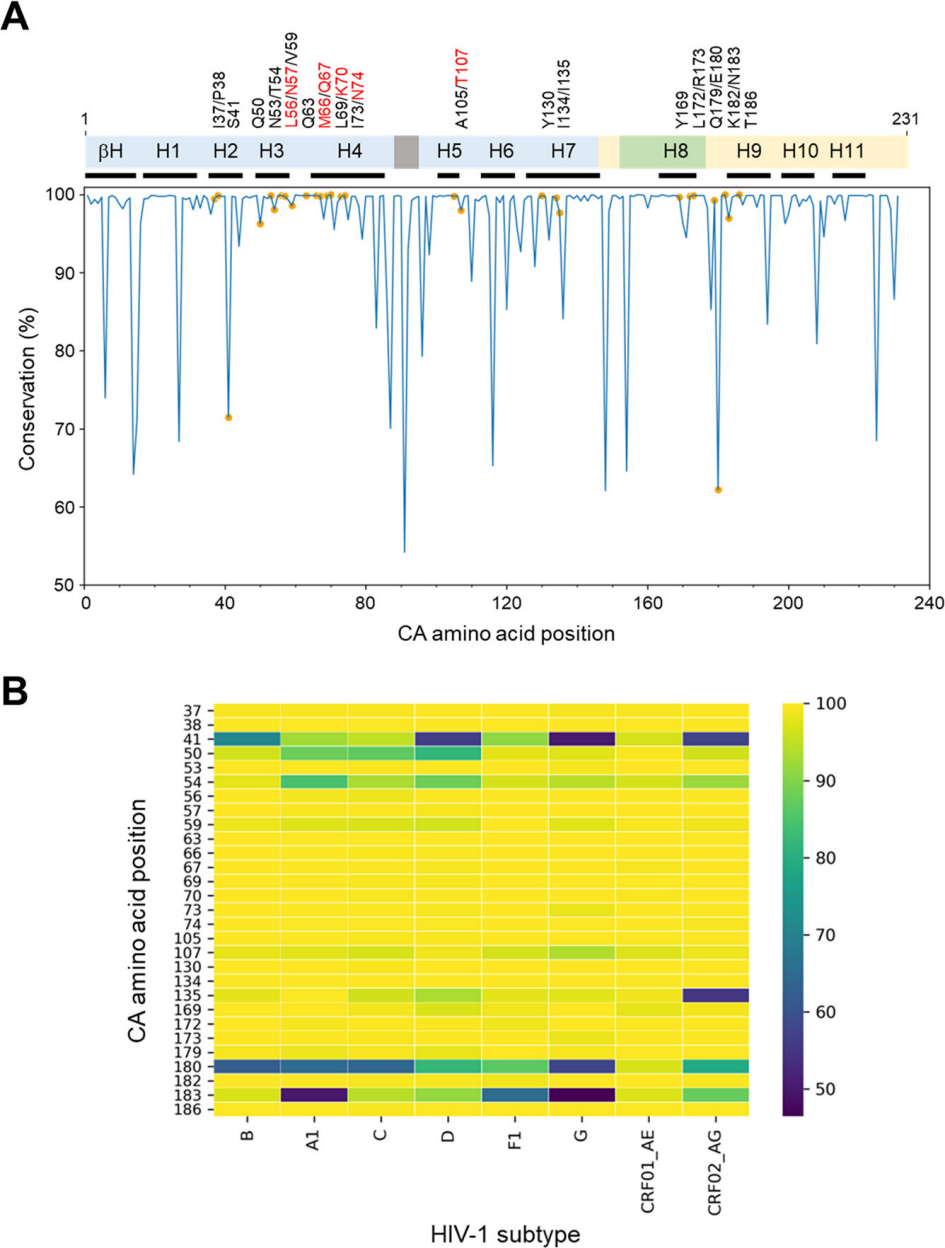
HIV-1 CA sequence conservation in clinical isolates. (A) Percent CA amino acid identity among 5,689 unique HIV-1 subtype B sequences relative to HIV-1 HXB2 reference sequence. Top, schematic illustrating the organization of a CA monomer (amino acids 1–231) composed of an N-terminal domain (NTD, blue), C-terminal domain (CTD, yellow), beta-hairpin (bH), alpha-helices (H1-11, individual boundaries denoted by lower black bars), cyclophilin A binding loop (gray), and major homology region (MHR, green). The 29 CA amino acids identified within five angstroms of LEN at its binding site are denoted above, with those previously implicated in the loss of LEN susceptibility highlighted in red. Bottom, percent CA sequence conservation plot with orange circles highlighting locations of LEN binding site residues. (B) Heat map depicting percent conservation of LEN CA-binding site residues by HIV-1 subtype. The number of unique sequences analyzed for each subtype is shown in Table 1. CRF, circulating recombinant form.

To investigate CA sequence diversity observed across the combined *gag* sequence data set, sequences were aligned to HIV-1 HXB2 (subtype B) reference sequence, and the conservation for each of the 231 amino acids in CA was calculated for each of the eight subtypes included in this study. Overall, 64.9% (150/231) of all CA residues were conserved (defined as ≤0.5% variability across all sequences) among subtype B isolates with a mean percent residue conservation across the entire CA protein of 96.8% (range 54%–100%) as shown in Fig. 2A, with all 29 CA residues identified within proximity to the LEN-binding site denoted as orange circles. The percentage of CA residues that were conserved for HIV-1 non-B subtype isolates analyzed here (A1, C, D, F1, G, CRF01_AE, and CRF02_AG) was also similarly high at 46.3% (107/231), 48.1% (111/231), 55.4% (128/231), 57.1% (132/231), 53.1% (123/231), 64.9% (150/231), and 53.2% (123/231) with mean percent residue conservation values across the entire CA protein of 94.8% (range 32%–100%), 95.9% (range 45%–100%), 96.4% (range 37%–100%), 96.4% (range 49%–100%), 95.7% (range 39%–100%), 97.3% (range 48%–100%), and 95.9% (range 38%–100%), respectively (Fig. S1).

### Identification of CA polymorphisms within the LEN-binding site

To compare the cross-clade percent amino acid conservation in CA specifically at the 29 residues of interest, a heatmap analysis was conducted and indicated a low degree of CA sequence variation in most LEN-binding site residues (Fig. 2B). A total of 18 of the 29 capsid residues with the potential to impact LEN binding (I37, S41, Q50, N53, T54, L56, N57, V59, I73, T107, I135, Y169, L172, R173, Q179, E180, K182, and N183) showed one or more substitutions above the 0.5% frequency threshold across the eight HIV-1 subtypes analyzed, for a total of 54 individual capsid variants detected across these positions (Table 2). Among these 18 polymorphic CA residues, five accounted for 59% of all amino acid substitutions detected with 11%, 13%, 13%, 5.6%, and 17% occurring at serine 41 (S41), glutamine 50 (Q50), threonine 54 (T54), glutamate 180 (E180), and asparagine 183 (N183), respectively. The remaining 11 CA residues (P38, Q63, M66, Q67, L69, K70, N74, A105, I134, Y130, and T186) were invariant across all 10,057 sequences analyzed at a 0.5% frequency cutoff. Notably, five of these 11 invariant residues (M66, Q67, K70, N74, and A105) were previously linked to LEN resistance (29). Although they were below the 0.5% frequency cutoff employed herein, the identities and prevalence of all “ultra-low” (<0.5%) frequency CA variants at each of the eight known LEN drug resistance-associated positions (codons 56, 57, 66, 67, 70, 74, 105, and 107) were also defined and are detailed in Table S1, with the overall prevalence of all currently known LEN resistance-associated mutations equal to 0.51% (51/10,057) across the entire analyzed sequence data set. Among the 54 naturally occurring CA polymorphisms identified herein, a total of 41 unique combinations of two to five CA polymorphisms were also observed in at least 1% of sequences for any given subtype. Notably, none of the observed combinations included any bona fide LEN resistance-associated mutations, suggesting that their combination is likely to present a low probability of conferring LEN resistance. For this reason, further investigation of these combinations was considered outside the scope of this study.

**TABLE 2:** Prevalence of LEN-binding site variants in CA across HIV-1 subtypes

CA amino acid^*b*^	Known resistant variants^*c*^	New variants identified in this study	CA amino acid substitutions^*a*^ within HIV-1 group M subtypes (*N*)							
			B (5,689)	A1 (489)	C (1,558)	D (430)	F1 (89)	G (128)	CRF01_AE (1,328)	CRF02_AG (346)
I37		V and Y		V^0.6^				Y^0.8^		
S41		A, I, M, Q, T, and V	T^23^ A^4.3^	T^6.8^ A^0.6^	T^96^A^0.5^	T^39^ A^1.6^ I^1.2^ M^0.7^	T^5.6^ A^2.3^ V^1.1^	T^47^ Q^1.6^ V^0.8^	T^3.0^	T^43^
Q50		A, G, H, P, S, T, and Y	S^1.7^ G^0.8^ H^0.8^	G^4.4^ H^2.5^ S^1.5^ T^1.3^ Y^1.2^	S^4.6^ T^2.4^ G^1.5^ A^1.2^ H^1.2^ P^0.6^	G^6.1^ S^4.9^ A^2.8^ T^1.9^ H^1.6^	H^1.1^T^1.1^	G^0.8^ S^0.8^ T^0.8^		T^1.2^ H^0.9^ G^0.6^
N53		K						K^0.8^		
T54		A, I, L, M, S, V, and Y	L^0.7^	M^85^ V^3.9^ L^0.8^	S^5.0^ A^0.6^	S^4.0^ M^3.3^ L^1.6^ V^1.4^	S^2.3^ I^1.2^	S^2.4^ L^1.6^ I^0.8^ Y^0.8^	M^97^ V^0.7^	M^92^ V^3.5^ L^1.2^ A^0.9^
L56	I	F, M, and V*		M^0.8^	F^0.6^					V^0.30*^
N57	S	H*				H^0.47*^				
V59		I	I^1.2^	I^2.3^	I^2.8^	I^3.8^		I^1.6^		I^1.2^
I73		F						F^0.8^		
T107	N	A, S, and V	S^1.5^	A^1.8^ S^0.6^	A^1.2^ S^1.2^	S^0.7^	S^2.3^ A^1.1^	A^3.9^ S^2.3^	S^2.6^	S^0.9^ V^0.6^
I135		H and V	V^2.2^		V^3.5^	V^6.8^	H^1.1^ V^1.1^	V^2.4^	V^1.1^	V^56^
Y169		F		F^100^	F^99^	F^2.8^	F^99^	F^100^	F^1.6^	F^99^
L172		V					V^1.1^			
R173		K						K^0.8^		
Q179		A, P, and T		A^0.8^ P^0.6^				T^0.8^		
E180		A, D, and P	D^38^	D^34^ A^0.6^	D^65^	D^83^	D^14^	D^43^	D^2.9^	D^20^P^0.9^
K182		R					R^1.1^			
N183		A, D, G, H, Q, R, S, T, and V	G^0.9^ T^1.1^ H^0.6^	G^53^S^4.7^H^2.3^ D^1.0^R^1.0^T^0.6^A^0.6^	G^2.7^ H^1.2^ S^0.6^	T^2.8^ G^2.6^ Q^0.9^ S^0.7^	G^65^ S^2.3^ A^1.1^ H^1.1^	G^40^ S^7.9^ H^2.4^ D^1.6^ V^0.8^ A^0.8^	H^1.7^	G^8.4^ S^1.7^ H^0.9^ A^0.6^

^*a*^ Unless indicated, only variants above a 0.5% cutoff prevalence are listed, with the percentage of prevalence noted in superscript values. Asterisks denote two variants initially identified from an interim analysis that was ultimately below the 0.5% prevalence cutoff.

^*b*^ According to HXB2 reference sequence. Eleven CA-binding site residues (P38, Q63, M66, Q67, L69, K70, N74, A105, I134, Y130, and T186) were invariant across these subtypes and are not shown.

^*c*^ Major CA inhibitor resistance-associated variants as defined by previous studies (29, 30, 51).

### Impact of CA polymorphisms on HIV infectivity

**Fig 3:**
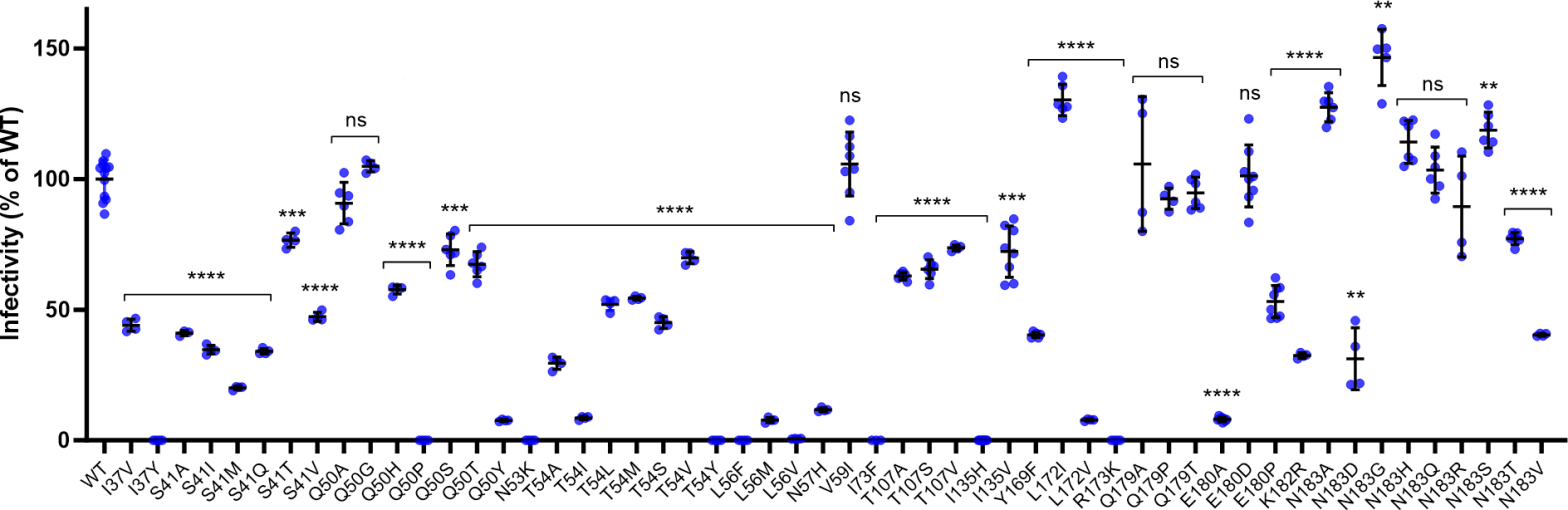
Infectivity of HIV-1 CA SDMs in MT-4 cells. WT and site-directed CA mutant single-cycle reporter HIV-1_NL4.3_ were produced in HEK293T cells by transient transfection, and the HIV content for each was determined by p24 antigen enzyme-linked immunosorbent assay (ELISA)using a single serial dilution of each sample and quantified across three samples within the linear range of the assay. MT-4 cells were infected in duplicate with serially diluted, p24-normalized WT and CA mutant viruses and developed 3 days later by One-Glo addition. Center line and error bars represent mean ± SD infectivity values, expressed as a percentage of the WT virus, obtained from at least three independent experiments. *P* values for each mutant (*n* = 3 replicate cell cultures for S41A and I73F mutants and 4–8 replicate cell cultures for all others) relative to the WT (*n* = 12 replicate cell cultures) determined by Brown-Forsythe and Welch ANOVA test. ****P < 0.0001; ***P < 0.001; **P < 0.01; ns, not significant (*P* > 0.05).

To evaluate the impact each CA polymorphism identified near the LEN-binding site has on viral infectivity, each of the 54 variants spanning 18 CA residues was individually introduced as a site-directed mutant (SDM) in the context of a single-cycle NL4.3-based reporter virus encoding firefly luciferase. The infectivity of each SDM was then evaluated in the MT-4 T-cell line relative to the p24-normalized WT control virus (Fig. 3). Of the 18 CA residues evaluated in this 54 mutant panel, seven residues (N53, N57, V59, I73, Y169, R173, and K182) were represented by a single polymorphism, three residues (I37, I135, and L172) were represented by two polymorphisms, and the remaining eight residues (S41, Q50, T54, L56, T107, Q179, E180, and N183) were represented by three or more polymorphisms detected above the 0.5% frequency cutoff. A majority of these CA SDMs (74%, 40/54) exhibited impaired HIV-1 infectivity (range 0.01%–77% of wild type), with the mean infectivity of I37Y, Q50P, N53K, T54Y, L56F, L56V, I73F, I135H, and R173K each being <1% of the WT control. Among the eight CA residues with three or more polymorphisms evaluated, amino acid substitutions at serine 41 (*n* = 6 variants), threonine 54 (*n* = 7 variants), and leucine 56 (*n* = 3 variants) were the least tolerated positions with mean infectivity values relative to the WT control virus of 42% (range 20%–77%), 37% (range 0.01%–70%), and 2.8% (range 0.06%–7.8%), respectively. In comparison, amino acid substitutions at glutamine 179 (*n* = 3 variants) and asparagine 183 (*n* = 9 variants) were generally well tolerated with mean infectivity values relative to the WT control virus of 98% (range 93%–106%) and 94% (range 31%–147%), respectively. Amino acid substitutions at glutamine 50 (*n* = 7 variants), T107 (*n* = 3 variants), and glutamate 180 (*n* = 3 variants) showed intermediate tolerability with mean infectivity values relative to the WT control virus of 57% (range 0.01%–105%), 67% (range 63%–74%), and 54% (range 8.1%–101%), respectively.

### Impact of CA polymorphisms on susceptibility to LEN

**Fig 4:**
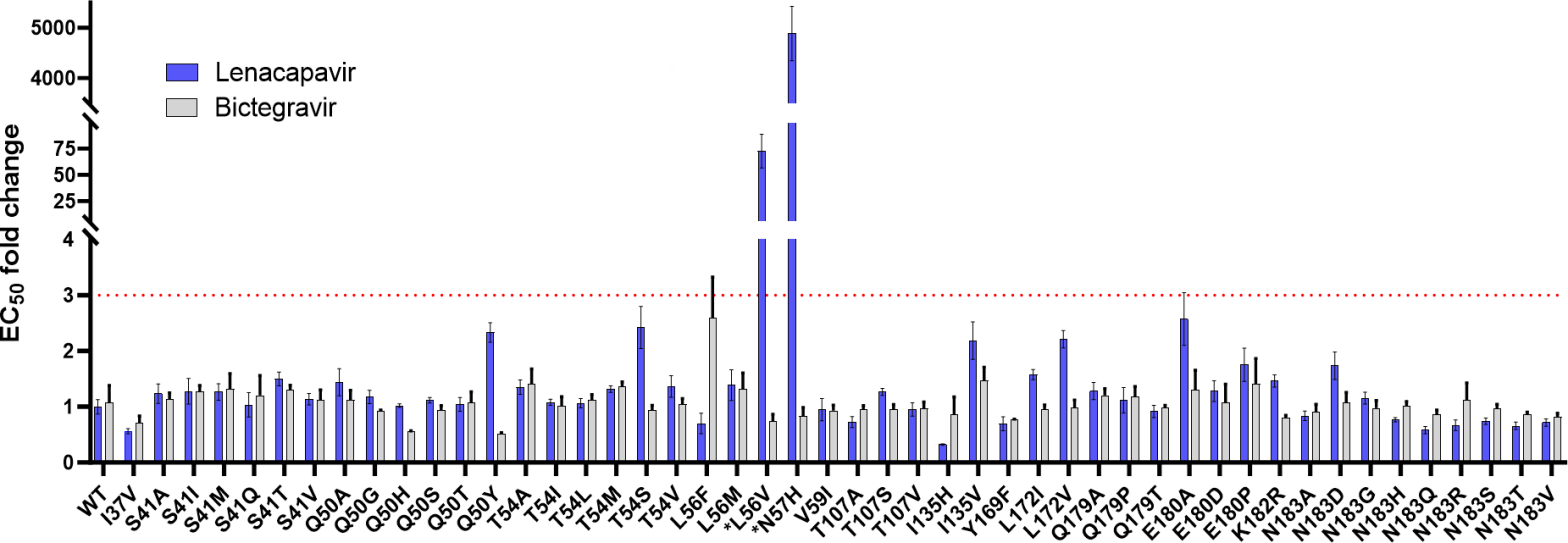
Fold resistance of single-cycle reporter HIV-1 strain NL4.3 encoding naturally occurring CA variants to LEN and a control antiretroviral agent (BIC) in MT-4 cells. Rectangles and error bars represent mean ± SD. EC_50_ FC values for each CA mutant relative to the WT virus from three independent experiments (*n* = 3 biological replicates each). The red dotted line defines the cut-off for drug resistance.

**Fig 5:**
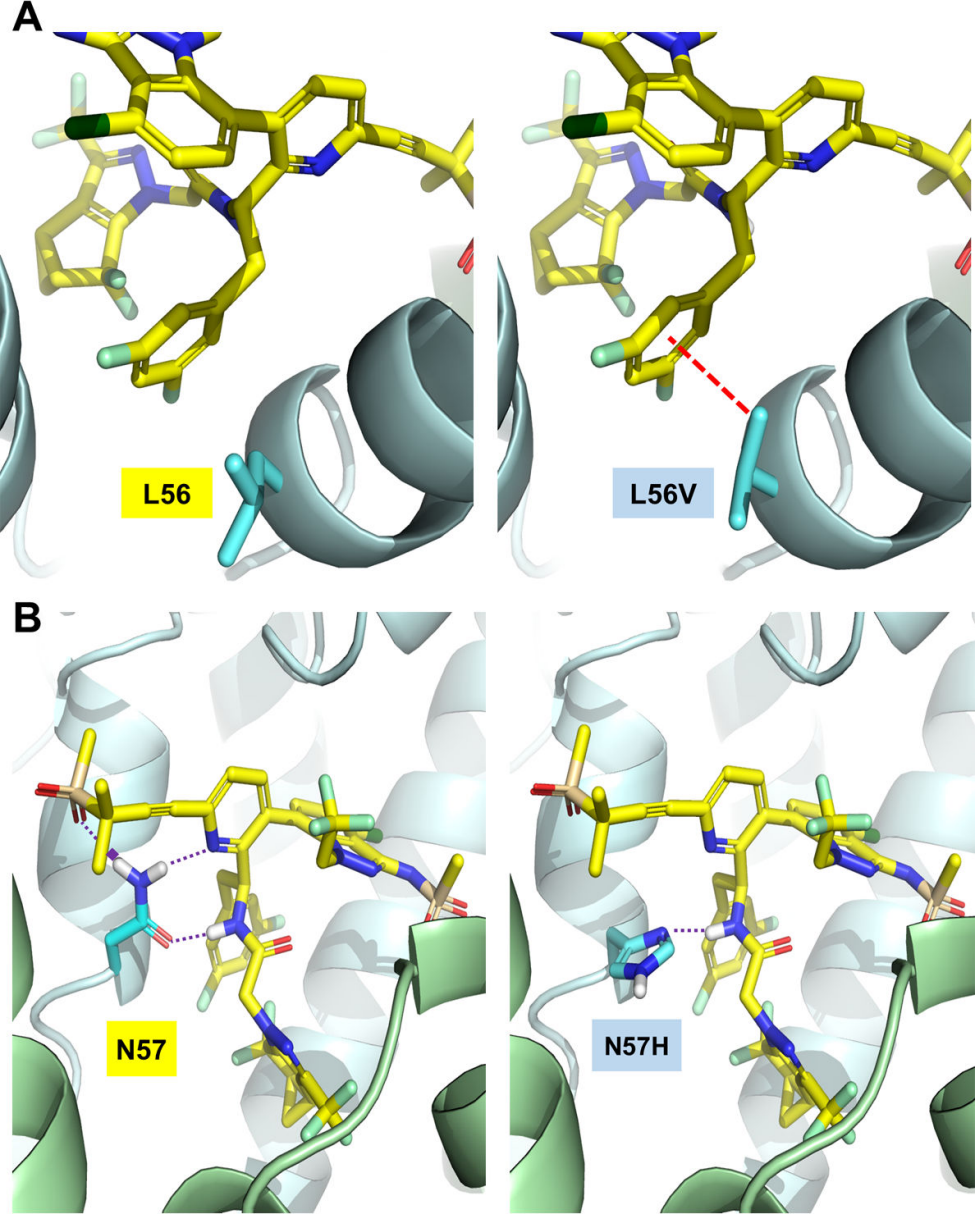
Structural impact of CA substitutions conferring loss of susceptibility to LEN. (A) Branching at the Cβ of the L56V mutation introduces steric clashing (red dashed line) with the central di-fluoro-benzyl group of LEN. (B) N57 forms three hydrogen bonds (purple dotted lines) with LEN, whereas the N57H variant forms only one.

To assess the impact these CA polymorphisms may have on HIV susceptibility to LEN, we determined the half-maximal effective concentration (EC_50_) value for LEN and bictegravir (BIC) as a control non-capsid targeting antiretroviral against WT and site-directed CA mutant HIV reporter viruses using a 3-day single-cycle antiviral assay conducted in MT-4 cells. Of the original 54 site-directed CA mutants produced, six variants (I37Y, Q50P, N53K, T54Y, I73F, and R173K) each showed a mean particle infectivity value so severely impaired (range 0.006%–0.03% of WT) that it precluded their phenotyping in our antiviral assay at any virus input tested. In comparison, the L56F, L56V, and I135H SDMs could each be reliably phenotyped using higher virus inputs despite also showing severely impaired particle infectivity (range 0.02%–0.60% of WT). Of the 48 CA variants exhibiting sufficient levels of particle infectivity to enable their phenotyping, 96% (46/48) remained fully susceptible to both LEN and BIC with mean EC_50_ fold change (FC) values for each mutant relative to the WT ranging from 0.6 to 2.6 for both inhibitors (Fig. 4). The remaining two CA variants, L56V and N57H, showed full susceptibility to BIC (FC values < 1 for each), but 72- and 4,890-fold reduced susceptibility to LEN, respectively. Both of these LEN-resistant CA variants were detected above our 0.5% prevalence cutoff in at least one subtype during an interim sequence analysis but later fell below that threshold at study completion, with final prevalences ranging from 0.30% to 0.47%. Structural modeling was performed to better understand the observed loss of susceptibility to LEN with each of these two rare CA variants (Fig. 5). The L56V substitution (0.6% WT infectivity) is predicted to interfere with LEN target site engagement by introducing steric clashing with LEN’s central di-fluoro-benzyl group, whereas the N57H substitution (12% WT infectivity) is predicted to lose two of three critical hydrogen bond interactions between WT asparagine 57 and LEN’s pyridine core, sulfone group, and amide linker.

### Impact of LEN-resistant CA polymorphisms on HIV-1 replication capacity

**Fig 6:**
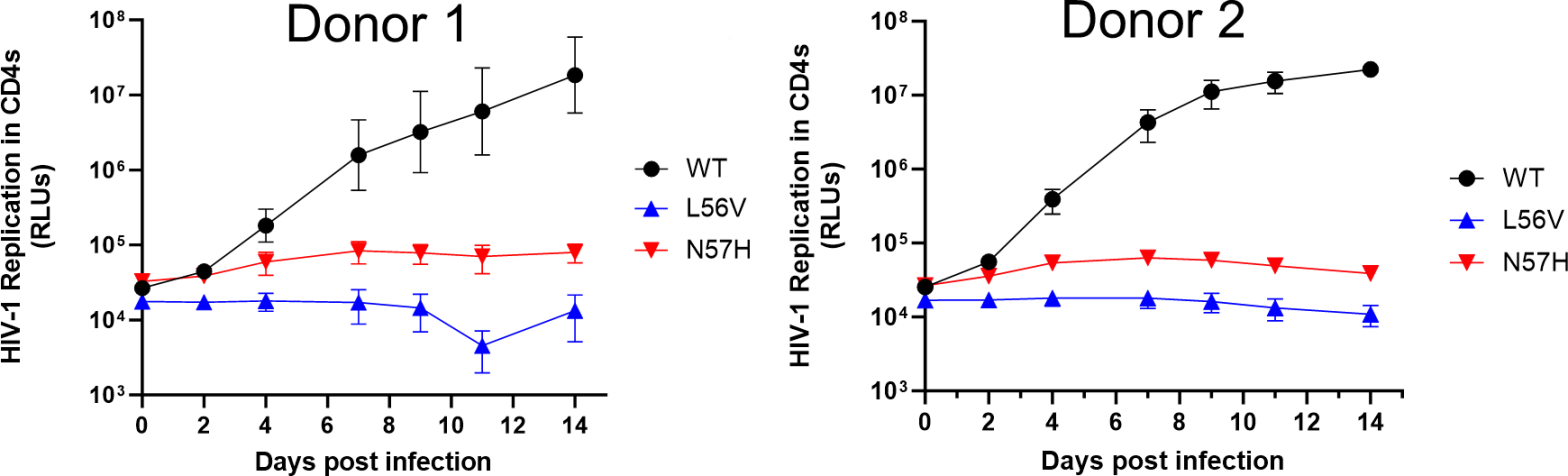
Replication competency of LEN-resistant naturally occurring CA variants. Outgrowth kinetics for replication-competent reporter HIV-1 encoding WT or each of the two LEN-resistant variants L56V and N57H in primary human CD4+ T cells obtained from each of two independent donors. Symbols and error bars represent mean ± SD luminescence values expressed relative to the WT virus from six replicate cell cultures in each of two independent experiments.

After observing that L56V and N57H single-cycle viruses confer high-level resistance to LEN but also reduced infectivity in MT-4 cells, we next investigated what impact these CA substitutions have on HIV-1 replication capacity in primary T cells. To do this, L56V and N57H were independently introduced into a replication-competent HIV-1 reporter virus (NL4.3 strain) expressing secreted NanoLuc luciferase. The outgrowth kinetics from p24-normalized inputs of these WT, L56V, and N57H viruses were then evaluated over a 14-day period in mitogen-activated primary human CD4^+^ T cells from two independent peripheral blood mononuclear cell (PBMC) donors by measuring the amount of secreted NanoLuc in cell-free supernatants obtained 0, 2, 4, 7, 9, 11, and 14 days post-infection (Fig. 6). While the WT virus supported a robust spreading infection with a nearly 3-log increase in secreted NanoLuc luciferase signal over the course of this 14-day viral outgrowth assay, the luciferase signal produced from the L56V and N57H variants remained largely unchanged (2.5-fold maximal signal increase) over the same time period, indicating that both of these LEN-resistant CA variants have a diminished capacity to establish a robust spreading infection.

## DISCUSSION

In this study, we performed a genetic surveillance of eight major HIV-1 Group M subtypes (A1, B, C, D, F1, G, CRF01_AE, and CRF02_AG) to identify 54 naturally occurring capsid polymorphisms across 18/29 LEN-binding site residues and then tested each capsid substitution individually for its impact on HIV-1 infectivity and susceptibility to LEN. Our analysis of >10,000 unique *gag* sequences demonstrated a high degree of CA sequence conservation, with 11/29 LEN-binding site residues being conserved (<0.5% prevalence) across this data set, including five residues that when mutated were previously implicated in contributing either alone or in combination to reduced susceptibility to LEN (M66, Q67, K70, N74, and A105) (29).

Among the circulating HIV-1 strains analyzed in this report, our study indicates that the collective prevalence of all currently known LEN resistance-associated mutations combined (L56I/V, N57H/S, M66I/V, Q67H/Y, K70H/N/R, N74D/S, A105E/T, and T107N) is quite rare (0.51%, 51/10,057), with the Q67H variant being the most represented RAM at 0.12% of total sequences. Q67H is one of the most predominant capsid variants observed during *in vitro* selection experiments and in HIV-1-infected individuals treated with subtherapeutic LEN concentrations or periods of unintended functional LEN monotherapy, and its emergence is sufficient to confer sixfold reduced drug susceptibility (29, 48). Our findings are in close agreement with prior studies of 21,111 and 805 capsid sequences that showed a collective prevalence of LEN RAMs equal to 0.20% and 0.25%, respectively, with Q67H similarly being the dominant LEN-resistant variant observed in each case (54, 55). Our results are also largely consistent with the 0.65% collective prevalence reported among the 2,031 capsid sequences analyzed by Nka et al., although their study differed in their identification of K70R instead of Q67H as the dominant LEN-resistant variant at 0.34% of sequences analyzed (56). By itself, the accessory mutation K70R does not significantly impact susceptibility to either LEN (FC = 1.4) or its close analog GS-CA1 (FC = 2.3) but can enhance capsid inhibitor resistance in combination with Q67H (51, 57). In two additional related studies, no LEN RAMs were detected by Marcelin et al. among 1,500 unpublished sequences from France or above a 0.1% prevalence in a report by Tao et al. among 21,012 uncurated multiclade Group M capsid sequences obtained from GenBank (58, 59). Collectively, these surveillance studies present a consistently low prevalence (<1%) of LEN RAMs across HIV-1 clades among LEN-naive individuals.

Our report uniquely expands on prior surveillance studies by including infectivity, antiviral, and viral outgrowth assessments to directly investigate the impact of naturally occurring polymorphisms within the LEN-binding site on viral infectivity and LEN drug susceptibility. Most of the identified capsid polymorphisms evaluated here (74%, 40/54) showed impaired infectivity relative to the wild-type virus, suggesting a strong selective pressure against sequence variation at many capsid residues within the LEN-binding site. While a majority (54%, 29/54) of the capsid polymorphisms analyzed here mapped to S41, Q50, T54, and N183, none of these substitutions conferred any resistance to LEN. In addition, only two of the LEN-binding site variants tested, L56V (0.30% among CRF02_AG) and N57H (0.47% among subtype D), showed reduced antiviral susceptibility to LEN. Notably, these novel capsid variants also showed impaired viral replication capacity relative to the wild type, as similarly observed for the previously identified capsid inhibitor resistance-associated mutants L56I and N57S (51). Considering that LEN shares a capsid-binding site with multiple phenylalanine-glycine (FG)-motif-containing cellular cofactors essential for HIV replication, including those present in the cytoplasm (Sec24C), the nuclear pore (Nup153), and the nucleus (CPSF6), it is not unexpected that a portion of the evaluated binding site substitutions could also impair these and possibly other capsid-host interactions necessary for the productive infection of target cells (29, 34–39, 60). Indeed, prior work has demonstrated that recombinant HIV-1 with substitutions within the FG pocket, such as the related capsid variants N57S, N57A, and N57D, loses the ability to interact with Nup153 and CPSF6, resulting in impaired import of the capsid and its cargo into the nucleus of nondividing cells and diminished integration into transcriptionally active chromatin, respectively (61).

There are two potential limitations of our present work that should be taken into consideration. First, as with prior genetic surveillance studies that rely heavily on publicly available sequence databases (54, 56, 59), our study included an overrepresentation of subtype B sequences (~57%) relative to their global distribution (11%) due to the increased availability of diagnostic testing in economically developed countries where it remains the predominant subtype. Conversely, the first and third most prevalent worldwide HIV-1 subtype C and A strains were underrepresented in our sequence collection at 16% and 4.9% of total sequences as compared to 50% and 12% worldwide, respectively (53). Similarly, our analyses focused exclusively on the eight major HIV-1 group M clades due to a relative scarcity of existing sequences representing multiple additional circulating recombinant forms and non-M group variants (N, O, and P), and thus, our sequence data set may not be fully representative of the worldwide HIV-1 subtype distribution. Second, although our work, to the best of our knowledge, is the first surveillance study to directly assess the impact of naturally occurring CA polymorphisms on viral infectivity and LEN drug susceptibility, the evaluation of these substitutions was done individually as SDMs within a single clade B (NL4.3) viral context. Thus, our study cannot exclude the possibility that certain combinations of individual CA polymorphisms and/or within different B or non-B strains may produce distinct context-dependent effects on viral infectivity and/or LEN drug susceptibility than those detailed here (62).

Lenacapavir is a currently approved option for use as part of a combination antiretroviral treatment regimen in people living with multidrug-resistant HIV-1 and is rapidly advancing through multiple clinical trials (PURPOSE 1–5) as a promising twice-yearly injectable agent for the global prevention of HIV-1 infection in people who could benefit from PrEP. Considering the high sequence conservation of HIV-1 CA observed across clades, together with the exceptionally low prevalence of LEN-resistant polymorphisms and their tendency to negatively impact viral infectivity, our data suggest that existing natural HIV-1 sequence diversity should minimally impact LEN efficacy in treatment-naive individuals.

## MATERIALS AND METHODS

### HIV-1 capsid population sequence analysis

For the public data set, HIV-1 *gag* sequences were downloaded from the Los Alamos National Laboratory (www.lanl.gov) and then deduplicated by keeping the first sequence from those with the same patient ID, resulting in a curated, unique *gag* data set encoding HIV-1 capsid protein. These CA sequences were then aligned to the HIV-1 HXB2 reference sequence (GenBank accession K03455) using Mafft v7.394 (63) for mapping of positions for conservation analysis. For each sequence, the associated HIV-1 subtype was determined using BLAST-based searches against a curated set of reference sequences and subsequently confirmed using the REGA HIV-1 subtyping tool V3.46. For the clinical data set, viral RNA was isolated from patient plasma samples from Gilead clinical trials (GS-US-200-4072, GS-US-200-4334, GS-US-200-4625, GS-US-236-0128, GS-US-264-0110, GS-US-380-1489, GS-US-380-1490, GS-US-536-5816, CO-US-985-6117, AELIX-002, NCS-0083, and NCS-0133), and the *gag* region encoding CA was amplified and sequenced by either Monogram Biosciences, Inc. (South San Francisco, CA) or Seq-IT (Kaiserslautern, Germany). Each amplicon underwent library preparation, barcoding, and pooling prior to deep sequencing using the Illumina-MiSeq next-generation sequencing platform. All resulting demultiplexed FASTQ files were processed via a custom data analysis pipeline as detailed below.

### HIV-1 capsid deep sequence analysis

Deep sequencing results were evaluated using a multi-step analysis pipeline. Adapter sequences were first removed, and low-quality reads were excluded using Trimmomatic software V0.32. Contigs were then generated using Vicuna software and aligned to generate a primary de-novo assembly sequence. Alternative assembly sequences using contigs not fully represented in the primary assembly sequence were used for more sensitive alignment of viral subpopulations. Reads were then aligned to assembly sequences using the SMALT sequence aligner. Any aligned reads that overlapped with the genomic coordinates of amplification primers were clipped. Amino acid realignment was performed on reads containing in-frame insertions and deletions (indels). Frameshift indels were trimmed to exclude the region with an indel from further analysis. All aligned reads were then translated in-frame, and changes from the reference sequence were tabulated based on observed codons in aligned reads. Any amino acid observed above 15% of the viral population and any indel above 50% were reported. Consensus sequences were generated from nucleotide variant calls and subtyped via a BLAST search against a curated HIV-1 subtype reference sequence library.

### Structural analysis

Structural models were based on the x-ray crystal structure of LEN bound to capsid hexamer (PDB 6V2F) (29). Bioluminate (Schrödinger Release 2023–4, New York, NY) was used to prepare the structures for simulations, adding protons, optimizing hydrogen bond networks, and relieving clashes and strain via the Protein Preparation workflow. From this structure, 29 amino acid side chains were determined to lie within five angstroms of LEN. Bioluminate was also used to generate single mutations and evaluate their impact on LEN binding via the MM-GBSA Residue Scanning workflow (64).

### Plasmids

The HIV-1 plasmid pKS13Δenv, encoding firefly luciferase from an *env*-defective NL4.3 backbone, and the expression plasmid pHCMV-G, encoding the vesicular stomatitis virus G (VSV-G) envelope, have been previously described (65). The replication-competent HIV-1 reporter plasmid pNL4-3-JRFL-secNLuc, encoding secreted NanoLuc luciferase (secNLuc) from an NL4.3-based infectious molecular clone, has been previously described (51). Site-directed mutant capsid genes encoding naturally occurring LEN-binding site polymorphisms were synthesized at GenScript (Piscataway, NJ) and inserted into pKS13Δenv and pNL4-3-JRFL-secNLuc plasmids via *BssH*II–*Apa*I ligation. Recombinant DNA for each construct was prepared at Genewiz (South San Francisco, CA) and confirmed by both *Hind*III restriction gel digestion and extensive sequence analysis at Elim Biopharmaceuticals (Hayward, CA).

### Compounds

The HIV capsid inhibitor LEN and the integrase strand transfer inhibitor BIC were synthesized at Gilead Sciences (Foster City, CA). All drug stocks were prepared in 100% dimethyl sulfoxide (DMSO) and stored frozen at −20°C.

### Cell lines

HEK293T cells were obtained from the Gladstone Institute for Virology and Immunology (San Francisco, CA) and maintained at densities below 80% confluency in Dulbecco’s Modified Eagle Medium (Life Technologies) supplemented with 10% heat-inactivated fetal bovine serum (FBS), 100 units/mL penicillin, and 10 µg/mL streptomycin. Cells were passaged twice per week using a trypsin-EDTA solution (Thermo Fisher Scientific, Waltham, MA) to detach adherent cells from culture flasks. The human T-lymphoblastoid MT-4 cell line was obtained from the NIH AIDS Reagent Program (Germantown, MD). Cells were cultured in RPMI-1640 medium (Life Technologies, Grand Island, NY) supplemented with 10% heat-inactivated FBS, 100 units/mL penicillin, and 10 µg/mL streptomycin (complete RPMI cell culture media). Cells were passaged twice per week and kept at densities of less than 800,000 cells per mL.

### Primary cells

Leukopaks were purchased from AllCells (Alameda, CA) and were obtained from consenting healthy volunteers participating in an Institute Review Board approved donor program. Donors were negative for HIV-1, hepatitis B, and hepatitis C infections. Human PBMCs were isolated from fresh leukopaks by standard Ficoll-Hypaque (Amersham, Piscataway, NJ) gradient centrifugation technique and cryopreserved for long-term liquid nitrogen storage in 90% heat-inactivated FBS and 10% DMSO at a density of 5 × 10^7^ cells/mL. Following their rapid thawing in a 37°C water bath and resting overnight at 3 × 10^6^ per mL in complete RPMI cell culture medium, isolated CD4+ T lymphocytes were prepared and activated at 3 × 10^6^ per mL in complete RPMI medium supplemented with 1 µg/mL phytohemagglutinin (PHA, Remel, San Diego, CA) and 50 IU/mL recombinant human interleukin-2 (IL-2; Roche Diagnostics, Indianapolis, IN) for 66–72 hours in a humidified 37°C incubator with 5% CO_2_ as previously described (66).

### Human immunodeficiency virus type 1

Single-cycle reporter HIV-1 encoding firefly luciferase was made by co-transfecting HEK293T cells with a 4:1 molar ratio of pKS13Δenv (encoding WT or site-directed capsid mutants) and pHCMV-G using the Liopfectamine-2000 transfection reagent (Thermo Fisher Scientific) according to the manufacturer’s recommended protocol. Cell-free viral supernatants were collected 3 days post-transfection and stored frozen as single-use aliquots at −80°C until ready for use. Replication-competent reporter HIV-1 encoding secreted NanoLuc luciferase was made by transfecting HEK293T cells with pNL4.3-JRFL-secNLuc plasmids encoding WT or site-directed capsid mutants. Cell-free viral supernatants were collected 3 days post-transfection, pelleted over a 20% sucrose cushion by centrifugation for 2 hours at 28,000 rpm, resuspended in phosphate buffered saline (PBS), and then purified by OptiPrep gradient fractionation prior to freezing as single-use frozen aliquots at −80°C. The amount of HIV in each virus preparation (single and multicycle) was quantified by p24 antigen ELISA (Perkin Elmer).

### Viral titering and infectivity assays

The infectivity of all VSV(G)-pseudotyped reporter HIV-1 was measured in MT-4 cells using 12-point, threefold dilutions of each p24-normalized virus prepared in complete RPMI cell culture media. Briefly, 50 µL aliquots of serially diluted, p24-normalized WT and CA mutant viruses were each added in duplicate to the wells of white opaque flat-bottomed 96-well plates (Corning Life Sciences, Tewksbury, MA) containing 50 µL aliquots of an MT-4 cell suspension at 1 × 10^6^ per mL in complete RPMI cell culture media. Assay plates were maintained in a humidified 37°C incubator with 5% CO_2_ for 3 days, after which time 100 µL of One-Glo Luciferase Assay reagent (Promega) was added to each well of the assay plates, and the amount of chemiluminescence signal was measured using an EnVision Plate reader (Perkin Elmer). The infectivity of each virus was calculated from the mean relative luminescence units observed over the linear range of the sample serial dilution and expressed as a percentage of that of the WT control virus.

### Antiviral assays

MT-4 cells were suspended at 1 × 10^6^ cells per mL in complete RPMI cell culture medium, and 50 µL aliquots of this cell suspension (5 × 10^4^ cells) were added to the inner 60 wells of white opaque flat-bottomed 96-well assay plates. Test compounds were then dispensed in triplicate as 2×, threefold serial dilutions (eight concentrations) using a Tecan D300e digital liquid dispenser. Working stocks of wild-type and capsid mutant VSV(G)-pseudotyped HIV-1 reporter viruses were then prepared in complete RPMI cell culture media, and 50 µL aliquots were added to the inner 54 wells. On each assay plate, six wells were also mock-infected with vehicle (DMSO)-containing media as the uninfected, no drug controls. The final DMSO concentration in the assay was 0.5%. After 3 days in a humidified 37°C incubator with 5% CO_2_, 100 µL of ONE-Glo Luciferase Assay reagent was added to each well of the assay plate, and the resulting chemiluminescence was measured using an Envision plate reader. EC_50_ values were calculated from compound dose-response curves using XLfit 5.5.0.5 software from IDBS (Boston, MA) and a four-parameter, nonlinear regression model.

### HIV-1 replication kinetics in primary human CD4+ T cells

Mitogen-activated CD4+ T lymphocytes were suspended in complete RPMI cell culture media supplemented with 10 IU/mL IL-2 and 10 µg/mL DEAE-dextran (Sigma Aldrich, St. Louis, MO) and seeded at 3 × 10^5^ cells per well in evaporation control 96-well plates (Nunc Cat # 267556). Cell cultures (50 µL) were then spinfected at room temperature for 2 hours at 1,200 × *g* with 2 µL of p24-normalized inputs of NL4-3-JRFL-secNLuc reporter HIV-1 encoding either WT or site-directed mutant capsids. At the completion of each spinfection, the volume in each well was increased to 300 µL, and the assay plates were transferred to a humidified 37°C incubator with 5% CO_2_ for a period of 14 days. To maintain an active infection, an aliquot of freshly activated CD4+ T cells was added to each well after 7 days. At designated timepoints post-infection, a fixed volume of cell-free media from each well was diluted in a complete RPMI medium and mixed 1:1 with Nano-Glo Luciferase Assay reagent (Promega). The resulting chemiluminescence was measured using an Envision plate reader and analyzed in GraphPad Prism 10.1.2.

### Statistics

GraphPad Prism 10.1.2 was used for statistical analysis. The Brown-Forsythe and Welch ANOVA tests for the infectivity of each mutant relative to the wild type were used to determine significance. A *P* value < 0.05 was considered statistically significant.

## SUPPLEMENTAL MATERIAL

Fig. S1 mbio.00187-25-s0001.tif Percent CA amino acid conservation across HIV-1 group M non-B subtypes.

Supplemental Material mbio.00187-25-s0002.docx Legend to Fig. S1; Table S1.

ASM does not own the copyrights to Supplemental Material that may be linked to, or accessed through, an article. The authors have granted ASM a non-exclusive, world-wide license to publish the Supplemental Material files. Please contact the corresponding author directly for reuse.
