# Supplementary figures and images for: Impact of HIV-1 capsid polymorphisms on viral infectivity and susceptibility to lenacapavir
Source: mBio. 2025 Apr 17;16(5):e00187-25. doi: 10.1128/mbio.00187-25 (PMC12077089; doi:10.1128/mbio.00187-25)

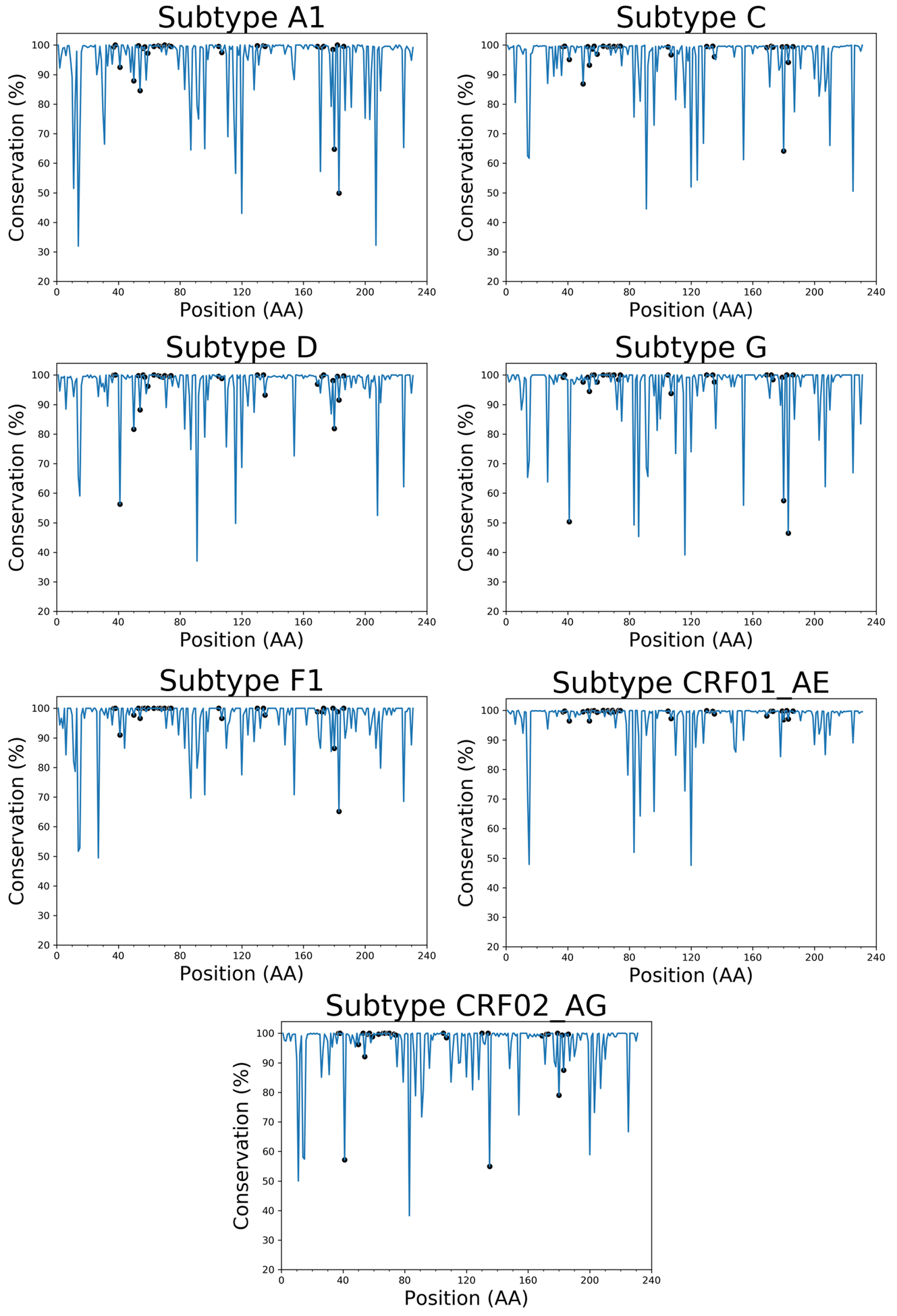

Supplement: Fig. S1 — Percent CA amino acid conservation across HIV-1 group M non-B subtypes. [file mbio.00187-25-s0001.tif]
