# Supplementary material for: Impact of HIV-1 capsid polymorphisms on viral infectivity and susceptibility to lenacapavir
Source: mBio. 2025 Apr 17;16(5):e00187-25. doi: 10.1128/mbio.00187-25 (PMC12077089; doi:10.1128/mbio.00187-25)
Supplement: Supplemental Material — Legend to Fig. S1; Table S1. [file mbio.00187-25-s0002.docx]

**Supplementary Materials**

**Figure S1. Percent CA amino acid (AA) conservation across HIV-1 group M non-B subtypes.** Conservation plot for each subtype is derived from curated HIV-1 *gag* sequences relative to HIV-1 HXB2 reference sequence, with black circles highlighting locations of LEN binding site residues. Unique sequences analyzed from subtypes A1 (n=489), C (n=1558), D (n=430), F1 (n=89), G (n=128), CRF01_AE (n=1328) and CRF02_AG (n=346) were downloaded from the Los Alamos National Laboratory ([www.lanl.gov](http://www.lanl.gov)) and supplemented with additional unique *gag* sequences obtained from Gilead-sponsored clinical trial samples from study participants never previously exposed to LEN.

**REFERENCES**

1. Tse WC, Link JO, Mulato A, Niedziela-Majka A, Rowe W, Somoza JR, Villasenor AG, Yant SR, Zhang JR, Zheng J. 2017. Discovery of Novel HIV Capsid Inhibitors with Long-Acting Potential. 24^th^ Conference on Retroviruses and Opportunistic Infections, abstract 38.
2. Yant SR, Mulato A, Hansen D, Tse WC, Niedziela-Majka A, Zhang JR, Stepan GJ, Jin D, Wong MH, Perreira JM, Singer E, Papalia GA, Hu EY, Zheng J, Lu B, Schroeder SD, Chou K, Ahmadyar S, Liclican A, Yu H, Novikov N, Paoli E, Gonik D, Ram RR, Hung M, McDougall WM, Brass AL, Sundquist WI, Cihlar T, Link JO. 2019. A Highly Potent Long-Acting Small-Molecule HIV-1 Capsid Inhibitor with Efficacy in a Humanized Mouse Model. Nat Med 25:1377-1384.
3. Link JO, Rhee MS, Tse WC, Zheng J, Somoza JR, Rowe W, Begley R, Chiu A, Mulato A, Hansen D, Singer E, Tsai LK, Bam RA, Chou CH, Canales E, Brizgys G, Zhang JR, Li J, Graupe M, Morganelli P, Liu Q, Wu Q, Halcomb RL, Saito RD, Schroeder SD, Lazerwith SE, Bondy S, Jin D, Hung M, Novikov N, Liu X, Villasenor AG, Cannizzaro CE, Hu EY, Anderson RL, Appleby TC, Lu B, Mwangi J, Liclican A, Niedziela-Majka A, Papalia GA, Wong MH, Leavitt SA, Xu Y, Koditek D, Stepan GJ, Yu H, Pagratis N, Clancy S, Ahmadyar S, et al. 2020. Clinical targeting of HIV capsid protein with a long-acting small molecule. Nature doi:10.1038/s41586-020-2443-1.
4. Bester SM, Wei G, Zhao H, Adu-Ampratwum D, Iqbal N, Courouble VV, Francis AC, Annamalai AS, Singh PK, Shkriabai N, Van Blerkom P, Morrison J, Poeschla EM, Engelman AN, Melikyan GB, Griffin PR, Fuchs JR, Asturias FJ, Kvaratskhelia M. 2020. Structural and mechanistic bases for a potent HIV-1 capsid inhibitor. Science 370:360-364.
5. Margot N, Pennetzdorfer N, Naik V, Rhee M, Callebaut C. 2023. Cross-resistance to entry inhibitors and lenacapavir resistance through Week 52 in study CAPELLA. Antivir Ther 28:1-5.

| **TABLE S1** | | | | | | | | | | |
| --- | --- | --- | --- | --- | --- | --- | --- | --- | --- | --- |
| **TABLE S1** Ultralow frequency capsid variants (<0.5% prevalence) at known LEN resistance-associated positions. | | | | | | | | | | |
| **Capsid​**  **Amino Acid^b^​** | **Known**  **resistant**  **variants^c^​** | **Capsid amino acid substitutions^a^ within HIV-1 group M subtypes (N)​** | | | | | | | | |
|  |  | **B​**  **(5,689)​** | **A1​**  **(489)​** | **C​**  **(1,558)​** | **D​**  **(430)​** | **F1​**  **(89)​** | **G​**  **(128)​** | **CRF01_AE​**  **(1,328)​** | **CRF02_AG​**  **(346)​** | **Total RAM incidence​**  **(10,057)​** |
| L56​ | I/V​ | A (1), **I (3)**, Q (1), **V (2)**, P (1)​ | A (1)​ | A (1), E (1), G (1), **V (2**)​ | -​ | -​ | -​ | K (1)​ | **V (1)**​ | L56I (n=3, 0.03%)​  L56V (n=5, 0.05%)​ |
| N57​ | H/S​ | K (3), Y (2), ​  **S (5)**​ | E (1), I (1), T (1)​ | D (1), G (1), **H (1),**K (1), M (2)​ | **H (2)**, Y (1)​ | -​ | -​ | K (1)​ | -​ | N57S (n=5, 0.05%)​  N57H (n=3, 0.03%)​ |
| M66​ | I/V​ | H (1), **I (2)**, L (1), **V (2)**​ | Y (1)​ | E (2), H (1), **I (1),**​  L (3), R (1), **V (1)**​ | S (1)​ | -​ | -​ | T (1)​ | -​ | M66I (n=3, 0.03%)​  M66V (n=3, 0.03%)​ |
| Q67​ | H/Y​ | A (1), E (2), **H (6)**, L (1), R (3)​ | A (1), E (1)​ | A (1), E (3), K (1)​ | **H (1)**, N (1)​ | -​ | -​ | **H (5)**, P (1), R (1)​ | -​ | Q67H (n=12, 0.12%)​  Q67Y (not detected, <0.01%)​ |
| K70​ | H/N/R​ | E (1)​ | ​ | E (4), I (1), **R (1)**, T (1)​ | **R (1)**​ | -​ | -​ | E (2), I (4), ​  **R (1)**​ | -​ | K70H (not detected, <0.01%)​  K70N (not detected, <0.01%)​  K70R (n=3, 0.02%)​ |
| N74​ | D/S​ | G (1), T (1)​ | **S (1)**, T (1)​ | A (10), L (2), ​  Q (1), **S (1)**​ | E (1)​ | -​ | -​ | K (1)​ | **D (1)**, Q (1)​ | N74D (n=1, 0.01%)​  N74S (n=2, 0.02%)​ |
| A105​ | E/T​ | **E (1)**, P (3), **T (4)**, I (1), S (2)​ | **T (2)**​ | **E (1)**, Q (2), R (1), S (1), **T (2)**, V (1)​ | P (1), V (1)​ | -​ | -​ | H (1), P (2)​ | -​ | A105E (n=2, 0.02%)​  A105T (n=8, 0.08%)​ |
| T107​ | N​ | G (1), I (4), P (1), R (1), C (1)​ | -​ | **N (1)**​ | -​ | -​ | -​ | C (1)​ | -​ | T107N (n=1, 0.01%)​ |
| a Variants below a 0.5% prevalence are listed, with the number of samples listed in parentheses. Amino acid substitutions associated with LEN-resistance are shown in boldface.  b According to HXB2 reference sequence. ​  c Major and accessory resistance-associated variants as defined by current and previous studies (Supplementary references 1-5). | | | | | | | | | | |
